# Transcriptome Phase Distribution Analysis Reveals Diurnal Regulated Biological Processes and Key Pathways in Rice Flag Leaves and Seedling Leaves

**DOI:** 10.1371/journal.pone.0017613

**Published:** 2011-03-02

**Authors:** Wenying Xu, Rendong Yang, Meina Li, Zhuo Xing, Wenqiang Yang, Guang Chen, Han Guo, Xiaojie Gong, Zhou Du, Zhenhai Zhang, Xingming Hu, Dong Wang, Qian Qian, Tai Wang, Zhen Su, Yongbiao Xue

**Affiliations:** 1 Laboratory of Molecular and Developmental Biology, Institute of Genetics and Developmental Biology, Chinese Academy of Sciences and National Centre for Plant Gene Research, Beijing, China; 2 State Key Laboratory of Plant Physiology and Biochemistry, College of Biological Sciences, China Agricultural University, Beijing, China; 3 State Key Laboratory of Rice Biology, China National Rice Research Institute, Chinese Academy of Agricultural Sciences, Hangzhou, China; 4 Research Center of Molecular and Developmental Biology, Key Laboratory of Photosynthesis and Environmental Molecular Physiology, Institute of Botany, Chinese Academy of Sciences, Beijing, China; Instituto de Biología Molecular y Celular de Plantas, Spain

## Abstract

Plant diurnal oscillation is a 24-hour period based variation. The correlation between diurnal genes and biological pathways was widely revealed by microarray analysis in different species. Rice (*Oryza sativa*) is the major food staple for about half of the world's population. The rice flag leaf is essential in providing photosynthates to the grain filling. However, there is still no comprehensive view about the diurnal transcriptome for rice leaves. In this study, we applied rice microarray to monitor the rhythmically expressed genes in rice seedling and flag leaves. We developed a new computational analysis approach and identified 6,266 (10.96%) diurnal probe sets in seedling leaves, 13,773 (24.08%) diurnal probe sets in flag leaves. About 65% of overall transcription factors were identified as flag leaf preferred. In seedling leaves, the peak of phase distribution was from 2:00am to 4:00am, whereas in flag leaves, the peak was from 8:00pm to 2:00am. The diurnal phase distribution analysis of gene ontology (GO) and cis-element enrichment indicated that, some important processes were waken by the light, such as photosynthesis and abiotic stimulus, while some genes related to the nuclear and ribosome involved processes were active mostly during the switch time of light to dark. The starch and sucrose metabolism pathway genes also showed diurnal phase. We conducted comparison analysis between Arabidopsis and rice leaf transcriptome throughout the diurnal cycle. In summary, our analysis approach is feasible for relatively unbiased identification of diurnal transcripts, efficiently detecting some special periodic patterns with non-sinusoidal periodic patterns. Compared to the rice flag leaves, the gene transcription levels of seedling leaves were relatively limited to the diurnal rhythm. Our comprehensive microarray analysis of seedling and flag leaves of rice provided an overview of the rice diurnal transcriptome and indicated some diurnal regulated biological processes and key functional pathways in rice.

## Introduction

A diurnal cycle is defined as a pattern that recurs over a 24-hr period. Plant diurnal oscillation is universal for plants and coordinates many biological pathways related to extracellular or intracellular signals, adapting the plants to daily alternation and maintaining a balance between metabolic reactions during light and darkness, especially for fluctuations of the carbon balance [1], [2], [3], [4], [5], [6], [7], [8], [9], [10]. During the light period, carbon fixation in leaves leads to sucrose synthesis through photosynthesis and the starch produced accumulates in the leaves. During darkness, the starch is degraded and so starch content decreases. Rice (*Oryza sativa*) is the major food staple for about half of the world's population and it is also a model monocot for studies of crop plants with relatively smaller genomes, due to the completion of its genome sequence. The rice genes related to starch synthesis are essential to improving grain quality (such as eating and cooking quality). In rice, flag leaves play important role in providing photosynthates to the filling grain.

The diurnal cycle also coordinates the opening and closing of stomata, affecting transpiration and changing the water potential in leaves. The opening of stomata facilitates carbon dioxide uptake for photosynthesis during the light [11], [12], [13]. The light regulation is essential in the metabolic and physiological functions of plants during the diurnal periods. Light is also crucial to entrain the endogenous circadian clock, ensuring the precise cyclic expression of circadian-regulated genes during the day. The significant correlation between diurnally oscillating genes during the diurnal cycle and growth hormone-responsive genes was revealed mostly through microarray-based transcriptome analysis [14], [15]. The plant growth-hormone pathways tightly interact with light signalling and the diurnal cycle in the control of plant growth.

Microarray experiments are normally used to collect large-scale time-series data, monitor genome-wide gene expression, profile the changes in transcripts, and identify novel genes regulated in the diurnal cycling and circadian clock during a 24-hr period [16]. The first version of an Affymetrix Arabidopsis microarray (containing 11,521 Arabidopsis ESTs) was applied to study the circadian clock-regulated key pathways in Arabidopsis by Harmer in 2000 [17]. In 2001, Ellen Wisman's group used another platform of microarray and found about 11% of the Arabidopsis genes showed a diurnal expression pattern and about 2% with a circadian rhythm [18]. Then the ATH1 Arabidopsis whole-genome array was also used to analyze the transcriptome throughout the diurnal cycle for clues concerning the diurnal and circadian-regulated starch metabolism in Arabidopsis leaves [1]. The transcriptome analysis for the diurnal changes of the starch metabolism-related genes indicated that there was transcriptional and posttranscriptional regulation of starch metabolism in Arabidopsis leaves. Recently, large microarray datasets related to the plant diurnal cycle (e.g. diurnal and circadian microarray data for Arabidopsis [1], [15], [18], [19], maize [20], [21], [22], barley [23] and soybean [24]) were published and some are available in public databases. In addition, the database of DIURNAL project (http://diurnal.cgrb.oregonstate.edu/) provides web-based data-mining tools that are multiple user-friendly for searching the diurnal and circadian microarray data of Arabidopsis, rice and poplar [19], [25].

During statistical analysis of diurnal microarray data, there are many challenges to correctly identify the subset of genes with a clear diurnal signature. A variety of methods have been developed to extract the cycling-expressed genes from microarray data for diurnal, circadian rhythm or cell cycle research. The computing methods mainly fall into two categories: frequency-domain and time-domain analyses [26].

The frequency-domain method is generally based on classical spectral analysis (e.g. Fourier transform and periodogram). After applying those methods, microarray expression profiles are transformed into frequency domains, then the rhythmic genes will yield the spectra that have a well-defined peak in the frequency domain. For diurnal or circadian-related genes, their periodogram will have a peak near a 24-hr period. Several prior studies have adapted these spectral methods to analyze biological data, e.g. FFT-NLLS [27], average periodogram [28] and Lomb–Scargle periodogram [29].

The time-domain method is an alternative method to analyze a time series using a pattern matching technique. To detect periodic patterns, the theoretical model is usually sinusoid. Two different methods are commonly used to measure the similarity between the models and the real data: nonlinear least-square curve fitting and cross correlation (CC). These algorithms assign to each time-series the properties of the model to which it is most similar. There are several programs available to perform the computation, including Cosinor [30], CORRCOS [17] and COSOPT [31]. Recently, we developed a new algorithm called ARSER to analyze diurnal or circadian expression data by combining the time-domain and frequency-domain analyses [32]. Testing by synthetic and real experimental data showed that it efficiently identified periodicity in short time-series.

Recently, little is known about the possible mechanism related to the rice diurnal cycle involved in metabolism, cellular function and growth. Global transcriptome analysis of the rice diurnal-cycle is also limited. In our study, we used the GeneChip Rice Genome Array representing 51,279 transcripts to monitor the expression profiles of rice flag leaves and seedling leaves during diurnal cycling. We employed the ARSER and CC methods together to analyze our microarray datasets. We also compared the predicted diurnal genes between rice and Arabidopsis. To elucidate the biological process involved in the rice day–night cycle, several approaches are being used, such as gene ontology (GO) enrichment analysis, MapMan, and cis-element analysis for the genes with similar diurnal phases. The present study might give some interesting insight into the light–dark diurnal cycle in plants.

## Results

### Experimental design and data quality

**Figure 1:**
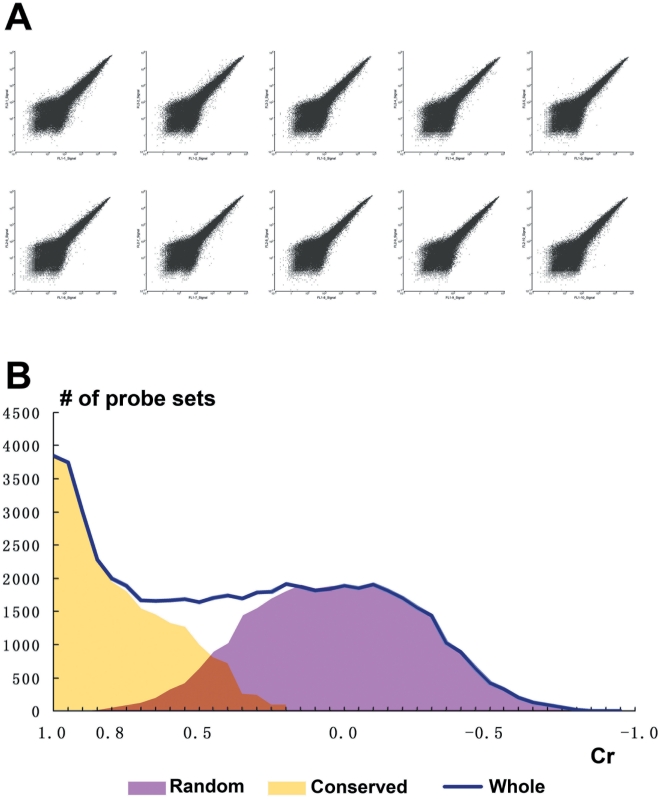
Expression profiles between the biological replicate samples of flag leaves. **A**: Pair-wise scatter plots for the raw probe-set intensity data across all time points. **B**: The number distribution of all probe sets based on the correlation coefficient (Cr) across the time series between the biological Os.7890.1.S1_x_at samples

Rice flag leaf and seedling leaf samples were collected every 4 hr in a period of 36 hr with two biological replicates. The expression profile for each sample was obtained by Affymetrix Rice Genome GeneChip analysis. Detailed pair-wise scatter plots of biological replicates were generated for flag leaves (Figure 1A) and for seedling leaves (Figure S1A). For biological replicates of each time point, nearly all of probe sets were fallen along the diagonal of plots, indicating no major variation. The correlation coefficients and false-positive rates of each pair of biological replicate samples were calculated by GCOS (Table 1). All correlations were >0.95, while false-positive rates were <5%. In summary, the data quality was satisfactory for identifying genes with diurnal patterns.

**Table 1:** Comparison of the expression profiles between the biological replicate samples.

Sample #	Time point (hr)	Seedling leaves		Flag leaves		Comments
		correlation	False Positive Rate (%)	correlation	False Positive Rate (%)	
1	8	0.98	0.85	0.98	3.82	Day 1 8:00am, the end of night
2	12	0.99	1.26	0.98	3.05	
3	16	0.99	1.18	0.98	2.21	
4	20	0.99	0.92	0.98	2.08	*Day 1 8:00pm*, *the start of night*
5	24	0.98	0.95	0.96	4.71	*Day 2 0:00am*
6	28	0.98	1.00	0.96	3.64	
7	32	0.98	1.00	0.99	1.62	*Day 2 8:00am*, *the end of night*
8	36	0.99	1.20	0.99	1.72	
9	40	0.96	4.09	0.99	1.50	
10	44	0.99	1.57	0.98	2.55	Day 2 8:00pm, the start of night

In addition, we calculated the correlation coefficient of each probe set (Cr) across the time-series of two sets of biological replicates in flag leaves and seedling leaves (Figure 1B and Figure S1B, respectively). As we had assumed, there were two forms among the expression patterns of each probe set along the detection time points: one was consistent and similar between biological replicates, with the Cr of replicate time-series tending to 1; the other was random, occasionally matched in biological replicates, and the average of Cr tended to 0. The number distribution of Cr (flag or seedling leaves) could be divided into two parts during analysis: the random part, close to a normal distribution, shown in purple in Figure 1B and Figure S1B; and the conserved part, shown in yellow, which represented the probe sets with consistent expression pattern.

In the meanwhile, we performed real-time RT-PCR to validate microarray results of flag leaves (Figure S2A) and seedling leaves (Figure S2B) in seven time points from 12 hr to 36 hr. Several genes were selected, including light-harvesting chlorophyll a/b binding protein (*LOC_Os03g39610*), starch and sucrose metabolism pathway related proteins such as glycogen/starch synthases (*LOC_Os07g22930*), 4-alpha-glucanotransferase (*LOC_Os07g46790*), and Glucan water dikinase (*LOC_Os06g30310*).The real-time RT-PCR results mostly matched the microarray expression patterns.

### Algorithms selected for diurnal pattern identification

To identify the genes with periodic expression patterns, we assumed that the expression profile of a gene exhibiting a diurnal pattern approximated a cosine wave with a period of nearly 24 hr. Several methods or algorithms are available for diurnal pattern identification, and two were selected: ARSER and CC. ARSER is a newly developed algorithm to analyze diurnal or circadian expression data by combining the time-domain and frequency-domain analyses, and was shown to be efficient in identifying periodicity in short time-series [32]. CC calculates the Pearson's correlation between a rhythmically-expressed gene and a theoretical cosine wave with a defined 24-hr period, and is a typical pattern matching technique [17].

**Figure 2:**
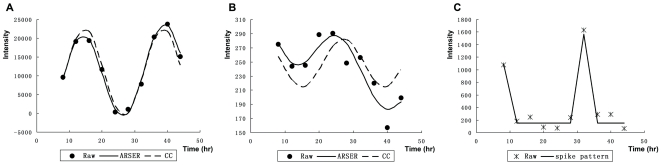
Examples for diurnal probe sets for different pattern identification algorithms. The scatter plot is a raw expression value of probe sets for 8–44 h, with sampling every 4 h; the solid line represents the cosine-curve model with ARSER (AR) simulation; the dash line represent the cosine-curve model with cross-correlation (CC) simulation. **A**: Os.7890.1.S1_x_at, seedling leaves, replicate sample 1. **B**: Os.15803.1.S1_at, seedling leaves, replicate sample 2. **C**: Os.22928.2.S1_at, flag leaves, replicate sample 1

Figure 2 shows some probe set examples with a diurnal pattern identified. Figure 2A presents the Os.7890.1.S1_x_at (*CAB* gene) expression value during 8–44 hr in 4 hr spaces with its predicted cosine-curve model using ARSER (AR) and CC methods. The calculation showed that the two methods almost agreed. The ARSER method gave a better fit than CC for the probe set for Os.15803.1.S1_at (Figure 2B). The probe set for Os.22928.2.S1_at showed a typical spike diurnal pattern (Figure 2C), which was only recognized by the ARSER method.

### The rice diurnal pattern genes in flag and seedling leaves

**Figure 3:**
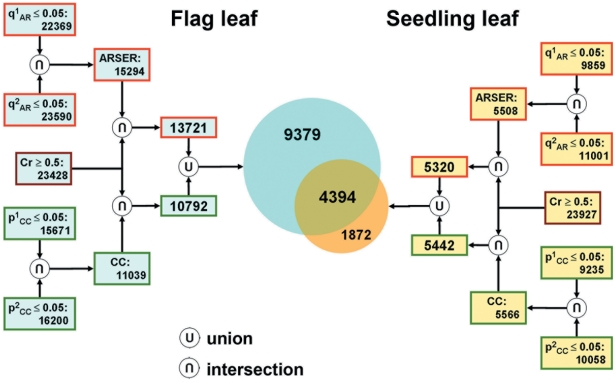
Identify the probe sets with diurnal pattern between flag leaves and seedling leaves. The orange shape indicates the diurnal-pattern probe sets only in seedling leaves, the light blue shape indicates the diurnal-pattern probe sets only in flag leaves, and the brown shape indicates the diurnal-pattern probe sets both in seedling and flag leaves.

The cosine-curve model of each probe set was calculated by the ARSER and CC algorithms, for every replicate sample series of flag and seedling leaves. To obtain more reliable diurnal pattern genes, we considered the p- or q-value of the model curve and the Cr between biological replicate sample series for each probe set. If the p- or q-values were all <0.05 in biological replicates and the Cr >0.5, then we used the combined results of the two algorithms. With the combinative criteria (Figure 3), 6,266 probe sets (10.96%) were identified with diurnal patterns in seedling leaves and 13,773 (24.08%) in flag leaves. The detailed information of these probe sets included raw intensity, cosine-curve model parameters, and gene annotation were shown in File S1. Within these diurnal pattern probe sets, there were 4,394 probe sets identified with diurnal patterns in both seedling and flag leaves, and 9,379 showed diurnal patterns that were preferred in flag leaves (Figure 3).

### The phase distribution of rice diurnal pattern genes in seedling and flag leaves

**Figure 4:**
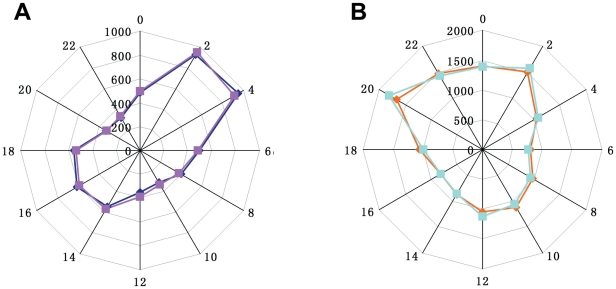
The phase distribution of the diurnal pattern probe sets in seedling leaves and flag leaves. **A.** The phase distribution of diurnal-pattern probe sets in seedling leaves: the dark blue indicates seedling leaf sample replicate 1, and the purple indicates seedling leaf sample replicate 2. **B**.The phase distribution of diurnal-pattern probe sets in flag leaves: the yellow indicates flag leaf sample replicate 1, and the light blue indicates flag leaf sample replicate 2

To further analyze the rice diurnal-pattern genes in seedling and flag leaves, all these genes were disassembled based on their phase of the cosine-curve model. The distribution of these probe sets in seedling and flag leaves are shown in Figure 4A and 4B, respectively. The distribution in two biological replicates was almost identical. For seedling leaves, the phases were during 2:00 am to 4:00 am, whereas in flag leaves the phases were during 8:00 pm to 2:00 am.

**Figure 5:**
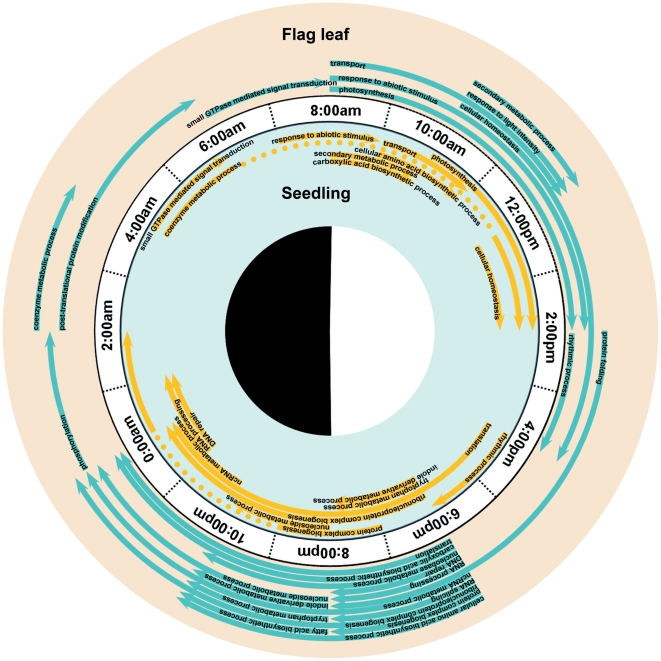
The Gene Ontology (GO) distribution along with the phase of the diurnal pattern probe-sets in seedling leaves and flag leaves. The middle circle with time stamp indicates the day–night cycle, inside for GO term distribution of seedling leaves and outside for flag leaves. Each line arc indicates the start point and end point (with arrow sign) of one GO term, the dashed arc indicates the temporary break of the GO term. The name of each GO term is shown on the arc.

To elucidate the biological process of the rice diurnal-pattern genes involved, we employed gene ontology (GO) enrichment analysis [33] on the probe sets within each phase-period (Figure 5). In the enriched GO term distribution, along with the diurnal pattern phase, there were several interesting findings. Some important processes were induced by the light, such as photosynthesis, response to abiotic stimulus, transporters, and secondary metabolic processes. Some other biological processes related to the nucleus and ribosomes were active during the night: the transcriptional regulation-related processes such as RNA processing, RNA splicing and DNA repair, were enriched in the later afternoon and evening; circadian rhythm was enriched in late afternoon; and small GTPase-mediated signal transduction was highlighted in early morning. Some flag leaves' specifically enriched GO terms were also shown in Figure 5, such as fatty acid biosynthesis processes in the early evening, phosphorylation at midnight, and post-translational protein modification from night to early dawn.

Further, we predicted common motifs in the promoter regions (2 kb, 5′-upstream of the ATG) of the genes with the same diurnal pattern phase. The possible diurnal cis-regulatory elements were identified using the published Web-based program ELEMENT [25] (http://element.cgrb.oregonstate.edu/) for rice genes distributed over the different phases of the entire day (Table 2). There were several diurnal-related cis-elements with significant presence in seedling and flag leaves, including Morning Element (ME, *CCACAC*), Evening Element (EE, *TATC*), SORLIP2AT (*GGCCC* or *GGGCC*), DOFCOREZM (*AAAG*), midnight enriched telobox (TBX, *AACCCT*), Element II of Arabidopsis PCNA-2 (*GGCCCA*, *TGGGCC*, or *AGCCCA*), AtSR1 (CaMBP, *CGCG*), ARR1AT, EECCRCAH1 (*AAAATC*), and TATABOX5. In flag leaves, the ME combined with the G-box (CACGTG) was significantly enriched in morning-expressed genes. Some important motifs were only present in the promoters of the flag leaves' preferentially expressed genes, such as GCCcore, CGACG element, SURECOREATSULTR11 (*GAGAC*, G-box/ABRE (*CACGTG*), BIHD1OS and WRKY71OS (*ATGTCA*).

**Table 2:** Common motifs in the promoter regions of diurnal pattern genes in phases.

Element	Phase	*0*	*2*	*4*	*6*	8	10	12	14	16	18	*20*	*22*	Annotation
*TATC*	Flag leaf	√	√						√	√	√	√	√	Evening Element (EE)
	Seedling								√	√	√	√		
*GGCCCA*	Flag leaf	√	√	√	√	√	√			√	√	√	√	Element II ofArabidopsis PCNA-2
	Seedling	√	√	√	√									
*TGGGCC*	Flag leaf	√	√	√	√					√	√	√	√	Element II ofArabidopsis PCNA-2
	Seedling	√	√											
*AGCCCA*	Flag leaf										√	√	√	Element II ofArabidopsis PCNA-2
	Seedling													
*GCCCA*	Flag leaf	√	√	√	√	√	√			√	√	√	√	overrepresented inthe OxPhos promoter
	Seedling	√	√	√	√									
*GGCCC*	Flag leaf	√	√	√	√	√	√				√	√	√	SORLIP2AT
	Seedling	√	√	√	√									
*GGGCC*	Flag leaf	√	√	√	√	√	√			√	√	√	√	SORLIP2AT
	Seedling	√	√	√	√									
*GCCGCC*	Flag leaf	√										√		GCCCORE
	Seedling													
*AACCCT*	Flag leaf										√	√	√	TBX,overrepresentedaround midnight
	Seedling											√		
*GATAA*	Flag leaf	√	√	√	√	√							√	I-box; rbcSt;
	Seedling									√	√			
*CAAAAC*	Flag leaf				√	√					√	√		ANAERO4CONSENSUS
	Seedling													
*CGCTT*	Flag leaf											√		WINPSTPIIIK
	Seedling	√												
*AAAG*	Flag leaf	√	√	√	√	√	√	√	√		√	√	√	DOFCOREZM
	Seedling	√	√	√	√				√	√	√	√		
*CCACAC*	Flag leaf					√	√	√						morning element (ME)RBCSBOX2PS
	Seedling					√	√							
*CACGTG*	Flag leaf					√	√	√	√		√			G-box/ABRE
	Seedling													
*ACGTG*	Flag leaf					√	√	√	√	√	√			ACGTATERD1
	Seedling					√								
*GCCAC*	Flag leaf					√	√							SORLIP1AT
	Seedling				√	√	√							
*CGCG*	Flag leaf			√	√	√	√	√	√					AtSR1(CaMBP)
	Seedling			√	√	√								
*CGACG*	Flag leaf					√	√	√						CGACG element
	Seedling													
*GAAAAA*	Flag leaf			√	√	√	√	√	√	√				GT1CONSENSUS andGT1GMSCAM4
	Seedling				√	√				√	√	√		
*GAGAC*	Flag leaf				√	√	√	√						SURECOREATSULTR11
	Seedling													
*AAATAA*	Flag leaf					√	√							TATABOX5
	Seedling				√	√								
*AAAATC*	Flag leaf								√	√	√	√		ARR1AT and EECCRCAH1
	Seedling									√	√			
*ATGTCA*	Flag leaf										√	√		BIHD1OS and WRKY71OS
	Seedling													

Sign √ indicates the element significantly present in the promoters of the genes shown diurnal pattern in proper phase.

### Rice transcription factor gene families with diurnal patterns in flag and seedling leaves

There were large numbers of transcription factor genes with diurnal patterns in both seedling and flag leaves. Several gene families of transcription factors, such as EIL, ZIM and ZF-HD, showed a flag-leaf-preferred diurnal pattern. About 65% of overall transcription factors were flag leaf preferred (Table 3). There were 20 families with a higher proportion of flag-leaf-preferred diurnal pattern, five were >90%, eight were within 80 to <90%, another four within 70 to <80%, and three within 65 to <70%.

**Table 3:** The rice transcription factors families showing flag leaf prefer diurnal patterns.

TF family	Seedling	Flag leaf	Flag leaf prefer	%
zf-HD	0	8	8	100.00
EIL	0	7	7	100.00
ZIM	1	6	6	100.00
SNF2	5	18	17	94.44
ABI3VP1	1	10	9	90.00
TCP	3	8	7	87.50
SET	5	19	16	84.21
MYB	13	24	20	83.33
Alfin-like	1	6	5	83.33
Jumonji	3	6	5	83.33
WRKY	10	26	21	80.77
NAC	13	20	16	80.00
PHD	6	20	16	80.00
ARF	7	19	15	78.95
AUX/IAA	6	17	13	76.47
C2H2	20	30	22	73.33
HB	17	41	29	70.73
C3H	19	40	27	67.50
MADS	9	12	8	66.67
TUB	4	9	6	66.67
AP2-EREBP	20	40	26	65.00
HSF	7	17	11	64.71
bHLH	21	28	18	64.29
CCAAT-HAP	7	14	9	64.29
MYB-related	16	37	23	62.16
Trihelix	5	13	8	61.54
GRAS	10	15	9	60.00
OVERALL	383	747	490	65.60

### Diurnal patterns of starch and sucrose metabolism related genes in seedling and flag leaves

**Figure 6:**
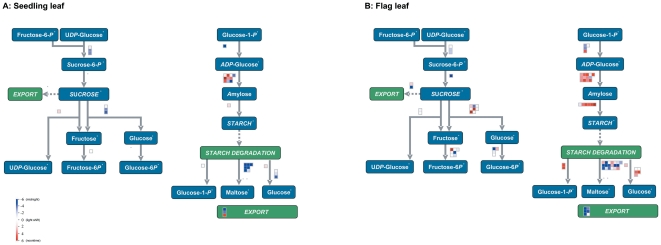
The diurnal pattern of key genes in starch and sucrose related pathway. The phase of each probe set was redefined as its relation to the time point of light shift (the start or the end of night). Red, blue and white squares to indicate the noon, midnight and light shift phase of the probe set, respectively.

The rice genes related to starch synthesis are essential to improving grain quality (such as eating and cooking quality). We searched the possible rice orthologs for the Arabidopsis starch-related genes, and made a rice gene-expression profile for the diurnal changes of sucrose metabolism-related enzymes in seedling and flag leaves, which were further displayed by MapMan (Version 3.0). We employed BLAST to map the probe sets to the BINs of MapMan, giving detailed mapping information of every probe set with a diurnal pattern (File S2). The phase distribution of the diurnal pattern probe-sets related to starch and sucrose biosynthesis and degradation pathways in seedling and flag leaves are shown in Figure 6A and 6B, respectively. There were similarities and a diversity of diurnal patterns between rice flag leaves and seedling leaves. The enzymes involved in the starch synthesis pathway mainly showed a daytime diurnal phase both in seedling and flag leaves. Overall, more probe sets with diurnal patterns occurred in flag leaves compared to seedling leaves: e.g. in the starch synthesis pathway, the majority of related genes showed a diurnal pattern during day time, both in seedling and flag leaves, but the genes encoding ADP-glucose pyrophosphorylases (AGPases) and starch-branching enzymes had significant diurnal patterns in flag leaves. Furthermore, the pathways derived from Tian et al. [34] showed diurnal patterns of individual enzymes in the starch synthesis pathway (Figure S3).

### Comparative analysis of rice and Arabidopsis diurnal pattern genes

There were similarities and differences between Arabidopsis and rice transcriptome analyses throughout the diurnal cycle. Recently, a large body of Arabidopsis expression profiling data related to diurnal or circadian rhythms was made publicly available. We selected the raw data (NCBI's Gene Expression Omnibus (GEO) accession number: GSE3416) from the study by Blasing [1] on diurnal gene expression of 5-6-week-old rosette leaves in *Arabidopsis thaliana* Col-0, and recalculated the diurnal pattern genes using the same approach, i.e. combining ARSER and CC. The detailed diurnal pattern Arabidopsis genes of GSE3416 are also listed in File S1. Thus, it is possible to compare the Arabidopsis diurnal pattern genes with those we identified in rice seedling leaves and flag leaves. Using the regular BLAST method, a close homolog between rice and Arabidopsis genes was mapped. Most diurnal-pattern genes in seedling and flag leaves had the Arabidopsis homolog (Table 4), and about half of these had a diurnal expression pattern. However, there were a large number of genes preferentially expressed with a diurnal pattern in rice flag leaves and seedling leaves that were not found in Arabidopsis leaves.

**Table 4:** Comparison between rice diurnal pattern genes in seedling leaves and flag leaves with Arabidopsis genes.

	Probe set ID	Match to Arabidopsis	Probe set ID (with Arabidopsis diurnal gene)	%
Diurnal in flag leaf	13773	11916	5868	49.24%
Diurnal in seedling	6266	5515	2792	50.63%
Overlap	4394	3942	2116	53.68%
Flag leaf prefer	9379	7974	3752	47.05%

**Figure 7:**
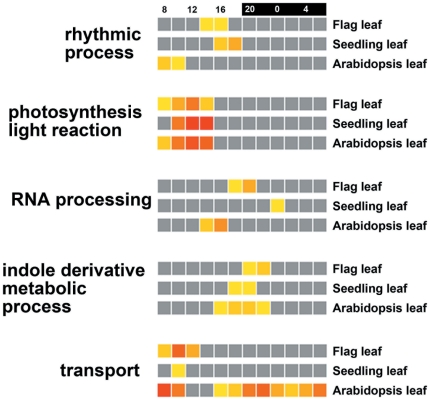
Selected GO term distribution along with the phase of the diurnal pattern between Arabidopsis and rice leaves. The comparison is displayed in HTML table mode for rice flag leaf, rice seedling leaf, and Arabidopsis leaf. The color blocks (yellow-to-red) represent the level of each term at a certain time-point. The greyscale represents non-significant enrichment.

Some phase specific diurnal cis-elements were compared between rice and Arabidopsis. The morning element, G-box, Evening Element, TBX and Element II of PCNA-2 were conserved and enriched in similar phases across rice and Arabidopsis. We also compared the GO term distribution between Arabidopsis and rice diurnal-pattern genes (Figure 7). Several processes such as photosynthesis and indole-derivative metabolic processes were similar between rice and Arabidopsis; however, many (e.g. rhythmic processes and RNA processing) were in different phases between the species.

## Discussion

### Identification of diurnal pattern genes in rice seedling and flag leaves

A variety of methods have been developed to extract the cycling-expressed genes from microarray data for circadian rhythm or diurnal research. The computational methods mainly fall into two categories: traditional time-series spectral analysis and cosine-based pattern matching methods. The advantages of the traditional spectral method are that they have been widely used to analyze time-series, and many programs are available for their calculation; however, their limitation is that they only perform well for long time-series. For microarray data, which usually have only a few points (due to high costs), the traditional spectral methods do not efficiently detect the exact diurnal or circadian rhythm period [35]. The cosine-based pattern matching methods are mathematically convenient with a reasonably good description of well-defined properties; however, they may not efficiently identify non-sinusoidal periodic patterns. Thus it is essential to use a suitable approach to identify the rhythmically-expressed genes during our analysis of diurnal microarray data.

The ARSER method [32] combines the time-domain and frequency-domain analyses and was shown efficient in identifying the periodicity in short time-series. Comparing with other cycling algorithms, ARSER can handle noise in the expression data and identify periodic patterns from limited sample-sizes and low numbers of replications of short time-series. Particularly, unlike cosine-curve-based algorithms, ARSER can identify both non-sinusoidal and sinusoidal patterns. For example, two transcripts with sinusoidal expression pattern can be identified as periodic by both ARSER and CC methods (Figure 2A and 2B), while one transcript with a non-sinusoidal (spike) expression pattern was identified as periodic by ARSER (Figure 2C). ARSER determines the statistically significant periodic transcripts by FDR q-values, which are calculated based on the distribution of p-values. The period range for computing will also impact on the selection of gene sets during the analysis of microarray data. By setting appropriate values for parameters, we could get more significant results using ARSER.

The combined use of ARSER and CC methods showed some special periodic patterns that were non-sinusoidal periodic patterns, such as probe set Os.22928.2.S1_at (Figure 2C) with a periodic expression value and a spike-shaped pattern according to HAYSTACK (http://haystack.cgrb.oregonstate.edu/). This approach appears to be efficient in analyzing short time-series compared with the spectral method and has a simpler computational procedure than for nonlinear curve fitting.

Combining the mathematical model and the reproducibility in biological replicates, we globally defined 6,266 (10.96%) diurnal probe-sets in seedling leaves and 13,773 (24.08%) in flag leaves. Transcriptome analysis showed that gene transcription levels in flag leaves were mostly of diurnal rhythm. Recently, a maize custom high-density 105 K Agilent microarray was conducted to investigate the diurnal expression patterns between the leaf and developing ears; about 22.7% (10,037) of expressed transcripts exhibited a diurnal cycling pattern in leaves, but only 0.39% (47) in developing ears [22]. These results indicated that the diurnal rhythms are related to developmental stages and tissue specificity, revealing a ‘third-dimension’ of diurnal rhythm regulation.

### Interactions between diurnal patterns and plant hormone signalling regulation in rice leaves

Light is a key environmental cue, and interactions among light, plant hormones and the circadian clock appear to control the diurnal patterns of plant growth. We analyzed the rice transcription factor families with flag-leaf-preferred diurnal patterns, and the results suggested that ethylene may also affect the diurnal pattern in rice flag leaves (Table 3). For example, four ethylene-insensitive3-like (*EIL*) genes (*LOC_Os07g48630*, *LOC_Os03g20780*, *LOC_Os03g20790* and *LOC_Os09g31400*) were identified as diurnal only in flag leaves but not seedling leaves. *EIL* may be involved in the ethylene signal-transduction pathway. Additionally, we also found that TIFY family proteins (recently discovered to play a critical role in repression of jasmonate signalling [36], [37], [38], [39], [40]), might also affect the rice diurnal pattern. Among 20 rice *TIFY* genes, only *OsTIFY11a* (*OsJAZ9*, *LOC_Os03g08310*) showed a diurnal pattern in seedling leaves, while another five showed a diurnal pattern in flag leaves, including *OsTIFY3* (*OsJAZ1*, *LOC_Os04g55920*), *OsTIFY6a* (*OsJAZ3*, *LOC_Os08g33160*), *OsTIFY6b* (*OsJAZ4*, *LOC_Os09g23660*), *OsTIFY10b* (*OsJAZ7*, *LOC_Os07g42370*) and *OsTIFY10c* (*OsJAZ8*, *LOC_Os09g26780*).

Rice diurnal microarray analysis showed that some abscisic acid (ABA)-dependent transcription factor genes (e.g. *ABI3VP1* and *OsNAC5*) had a significant diurnal pattern in rice flag leaves (Table 3). Common motifs such as CACGTG (G-box/ABRE), ACGTG (ABRELATERD1) and CACG (NAC core motif), were identified in the promoter regions (2 kb 5′-upstream from the ATG) of the genes with similar diurnal pattern peaks in the day time (from phase 8 to phase 18). In Arabidopsis, a large number of ABA-responsive and/or methyl jasmonate (MeJA)-responsive genes were identified with oscillation expression diurnally and robustly during the light–dark cycle [14], [15]. Plant stomatal movements are rhythmic and ABA can regulate the diurnal oscillator period [41], [42]. From motif analysis (Table 2), we also found that CGCG Box (calmodulin-binding) was enriched in the promoter regions of the diurnal genes with expression peaks from morning to noon (phases 6, 8, 10, 12 and 14). This may be related to the possible dynamic change of the concentration of internal calcium which oscillates diurnally, peaking during the day and dropping at night.

Auxin is a key regulator of plant growth and development, and auxin signal transduction can be regulated by the circadian clock in Arabidopsis [15], [43]. The plant sensitivity to auxin was observed to vary according to the time of day. Through microarray analysis, several AUX/IAA and ARF genes showed significant diurnal patterns in rice flag and seedling leaves. Through promoter analysis for the rice diurnal genes, we found that common motif ATGTCA/TGTCA [SURECOREATSULTR11, the core of sulfur-responsive element (SURE) which contains auxin response factor (ARF) binding sequence] was significantly enriched in the promoter regions of the diurnal genes in flag leaves, with expression peaks from morning to noon (phases 8, 10 and 12), while in seedling leaves the expression peaks were in the evening (phase 22).

From the diurnal genes in flag and seedling leaves, some gibberellin-mediated signalling and metabolic-related genes were enriched in seedling leaves, including ent-kaurene synthase A (*LOC_Os04g09900* and *LOC_Os02g36210*), ent-kaurene synthase B (*LOC_Os02g36140*, *LOC_Os04g10060*, *LOC_Os11g28530* and *LOC_Os12g30824*) and ent-kaurene oxidase (*LOC_Os06g37364*). Among the seven gibberellin-related genes, two (*LOC_Os12g30824* and *LOC_Os06g37364*) also peaked at midnight in flag leaves.

### Starch metabolism in rice diurnal cycling

During the diurnal cycle, starch is stored in leaves in a pattern such that starch content increases during periods of light and decreases during darkness [44], [45], [46]. Starch is one of the most important compounds synthesized by plants, and higher starch levels in leaves can lead to increased biomass. The carbon contained in leaves can be converted to ethanol for use as a bio-fuel and an alternate energy source. The genes related to starch synthesis are also essential for improving grain quality (e.g. eating and cooking quality). Our microarray-based diurnal gene identification showed similarities and diversity of diurnal patterns for starch metabolism-related genes between rice flag and seedling leaves (Figure 6 and Figure S3). The enzymes involved in the starch synthesis pathway mainly showed a daytime diurnal phase, both in seedling and flag leaves; whereas there were more genes of the starch synthesis enzymes with a diurnal pattern only in flag leaves (e.g. AGPase and SBE1). Our results showed no AGPases with a diurnal pattern in seedling leaves, but *LOC_Os09g12660* (the small subunit of AGPase) had a diurnal phase at 4:00pm in flag leaves. AGPase catalyzes the reaction generating the sugar nucleotide ADP-glucose and inorganic pyrophosphate from glucose 1-phosphate and ATP, which is the first step of starch biosynthesis. AGPase is considered as a major enzyme controlling starch synthesis [47]. Starch branching enzyme (SBE) acts on glucose polymers through α-1,6-glucosidic bonds to form branches on the α-1,4-linked glucose backbone, which is also a key enzyme in the starch biosynthesis pathway [48], [49], [50]. In sorghum endosperm, three SBE genes showed a diurnal rhythm in gene expression levels during a 24-h cycle [51]. Our result showed that four rice probe-sets matched *LOC_Os06g51084* (*SBE1*) with a diurnal pattern only in flag leaves. The starch metabolism genes with a diurnal pattern in flag leaves may play special roles during graining filling, beneficial to grain quantity and quality.

### Possible ribosome and chromatin related transcriptional regulation during light–dark diurnal cycle

Enriched GO term analysis for the assigned diurnal genes (Figure 5) showed that the genes related to the nuclear and ribosome-involved processes were active mostly during the period of light–dark change, which is similar to the GO phase distribution of diurnal transcripts in maize leaves [22]. This may be related to light-caused DNA mutations and DNA repair during the day and night. We also found that SNF2 family genes were significantly expressed with a diurnal pattern in flag leaves (Table 3). SNF2 is the catalytic subunit of the SWI/SNF chromatin remodelling complex and, SNF2-family genes play important roles in transcriptional regulation, maintenance of chromosome integrity and DNA repair [52], [53], [54], [55]. The Arabidopsis SWI2/SNF2 chromatin remodelling gene-family was reported to be involved in DNA damage response and recombination [53]. The diurnally-regulated SNF2 family genes may be related to changes in chromatin structure at the core of the diurnal oscillator, which may provide a clue concerning the regulation of diurnal progression by chromatin dynamics.

## Materials and Methods

### Plant materials

Seeds of rice (*Oryza sativa* subsp. *japonica* var. Nipponbare) were surface-sterilized in 5% (w/v) sodium hypochlorite for 20 min and then washed in distilled water three or four times, then germinated in water for 2 d at room temperature and 1 d at 37°C. The seedlings were transferred to water-saturated Whatman filter paper and grown in a greenhouse (28/25°C and 12/12 h of light/dark, and 83% relative humidity). After about 17 d, seedling leaves were harvested every 4 hours.

For flag leaf samples, the rice plants were grown in the field under natural conditions, within the May–October growing season, on an experimental farm in Zhejiang, China. Three-month-old rice plants were entrained into the greenhouse under the regular condition (32/30°C and 12/12 h of light/dark). Flag leaves were harvested every 4 hours.

### RNA isolation and Affymetrix GeneChip experiments

All leaf samples were homogenized in liquid nitrogen before isolation of RNA. Total RNA was isolated using TRIZOL® reagent (Invitrogen, CA, USA) and purified using Qiagen RNeasy columns (Qiagen, Hilden, Germany). For each sample, 8 µg of total RNA was used for making biotin-labelled cRNA targets; cDNA and cRNA synthesis; cRNA fragmentation, hybridization, washing and staining; and scanning, following the GeneChip Standard Protocol (Eukaryotic Target Preparation). In this experiment, a Poly-A RNA Control Kit and a One-Cycle cDNA Synthesis kit were applied. Affymetrix rice genome arrays were used for hybridizations.

### Real-time RT-PCR

Reverse transcription was performed using M-MLV kit (Invitrogen). We heated 10 µl samples containing 2 µg of total RNA, and 20 pmol of random hexamers (Invitrogen) at 70°C for 10 minutes to denature the RNA, and then chilled the samples on ice for 2 min. We added reaction buffer and M-MLV enzyme to a total volume of 20 µl containing 500 µM dNTPs, 50 mM Tris–HCl (pH 8.3), 75 mM KCl, 3 mM MgCl_2_, 5 mM dithiothreitol, 200 units of M-MLV, and 20 pmol random hexamers. The samples were then heated at 37°C for 1 h. The cDNA samples were diluted to 8 ng/µl for real-time RT-PCR analysis.

For real-time RT-PCR, triplicate assays were performed on 1 µl of each cDNA dilution using the SYBR Green Master Mix (Applied Biosystems, PN 4309155) with an ABI 7900 sequence detection system according to the manufacture's protocol (Applied Biosystems). The gene-specific primers were designed by using PRIMER3 (http://frodo.wi.mit.edu/primer3/input.htm). The amplification of 18S rRNA was used as an internal control to normalize all data (forward primer, 5′-CGGCTACCACATCCAAGGAA-3′; reverse primer, 5′- TGTCACTACCTCCCCGTGTCA-3′). The primer sets of four selected genes were listed below: *LOC_Os03g39610* (5′-ATGTTCTCCATGTTCGGCTTCT-3′ and 5′-GTATGCCCAGGCGTTGTTG-3′), *LOC_Os07g22930* (5′-GACGTCACAGCGGTTGCTT-3′ and 5′-TTCTTGGAATGCCGGTGTGT-3′), *LOC_Os07g46790* (5′-TGAAAACGAGGAGTGGTTGAAA-3′ and 5′-GGCCCCATTGGCTGTGA-3′), and *LOC_Os06g30310* (5′-TTACAGATCAGGGATTCCAAAAATC-3′ and 5′-ATAAAGCTCCTCAATGGCATGAC-3′). The relative quantification method (DDCT) was used to evaluate quantitative variation between replicates examined.

### Transcriptome data analysis and diurnal pattern identification

The signal intensity for each probe set on the GeneChip microarray was extracted by Affymetrix GCOS software and the TGT (target mean value) was scaled as 500 for each chip. Pair-wise scatter plots of replicate samples were generated by Partek Genomics Suite (Version 6.3). For each probe set, we calculated the correlation coefficient (Cr) of two sets of biological replicates across the time series.

Two methods were applied to identify diurnal pattern probe sets: ARSER and cross-correlation (CC). ARSER employs autoregressive spectral estimation to predict the periodicity of an expression pattern from the frequency spectrum and then models the rhythmic patterns using a harmonic regression model to fit the time-series [32]. There are four steps during the ARSER method: the ‘detrending process’, performs a data pre-processing strategy that removes any linear trend from the time-series; then autoregressive spectral analysis calculates the power spectral density of the time-series; further harmonic analysis provides the estimates of the parameters that describe the rhythmic patterns; and finally, false-discovery rate q-values are calculated for multiple comparisons. The CC method was used to calculate the Pearson's correlation between a rhythmically-expressed gene and a theoretical cosine wave with defined phase [17]. The brief calculation process follows. First, we used the cosine curve (Equation 1) with phases of 0–24 hr to prepare 60 test cosine-curves of 24-hr periodicity. The time span was 36 hr long with one and half cycle and interval between adjacent phases equal to 0.4 hr. Second, we calculated the C_α_ of the best-fitting cosine curve for each expression profile and the phase of the best-fitting cosine curve was defined as the phase of the related probe sets. Third, we used a random Monte Carlo simulation to determine the statistical significance p-value: we randomly produced 100,000 expression profiles, and calculated the maximum C_β_ for each of them. We then counted the number of times that a random expression profile showed C_β_ greater than a specified value and defined the p-value as the number divided by the number of all random expression profiles [56].



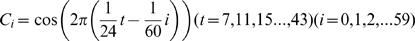
(1)


### Gene annotation, Gene Ontology analysis, and pathway analysis

The consensus sequence of each probe set was compared by BLAST (Basic Local Alignment and Search Tool) against the TIGR Rice Genome version 5 to map the probe set ID to the locus ID in the rice genome. The cut-off e-value was set as 1e–20. Within the 57,195 designed probe sets in the Affymetrix rice genome array, there are 52,697 probe-sets mapped to rice genes in TIGR rice pseudomolecules.

The promoter sequences were extracted from MSU Rice Genome Annotation Website (http://rice.plantbiology.msu.edu/) and ELEMENT (http://element.cgrb.oregonstate.edu/). ELEMENT and Place (http://www.dna.affrc.go.jp/PLACE/) [57] were used for motif search and cis-element identification.

The GO category enrichment analysis was applied by in-house agriGO analysis service [33], the Singular Enrichment Analysis (SEA) and Cross comparison of SEA (SEACOMPARE) tools with default parameters for the Affymetrix rice genome array used for analysis.

MapMan (http://gabi.rzpd.de/projects/MapMan) was used for key regulation group analysis. The starch biosynthesis pathway was adopted from that of Tian [34] and the corresponding MapMan pathways were created through the mapping files.

## Supporting Information

Figure S1 **Expression profiles between the biological replicate samples of seedling leaves.** **A**: Pair-wise scatter plots for the raw probe-set intensity data across all time points. **B**: The number distribution of all probe sets based on the correlation coefficient (Cr) across the time series between the biological replicate samples (including file name, file format, name and URL link of appropriate viewer if format is unusual).
(TIF)

Figure S2 **Real-time RT-PCR validation for selected genes.** Four genes (LOC_Os03g39610, LOC_Os07g22930, LOC_Os07g46790, and LOC_Os06g30310) were selected for real-time RT-PCR to validate the expression patterns of flag leaves (**A**) and seedling leaves (**B**) in seven time points from 12 hr to 36 hr. The purple and yellow bars represent the expression level in microarrys, and the blue bars represent the relative intensity of real-time RT-PCR from independent biological replicates.
(TIF)

Figure S3 **The diurnal pattern of the key genes in the starch biosynthesis pathway.** The phase of each probe set was redefined as its relation to the time point of light shift (the start or the end of night). Red, blue and white squares to indicate the noon, midnight and light shift phase of the probe set, respectively.
(TIF)

File S1 **Raw tables for diurnal probe-sets list.**
(XLS)

File S2 **Tables for diurnal probe sets related to sucrose and starch pathways**.
(XLS)
