# Supplementary figures and images for: Transcriptome Phase Distribution Analysis Reveals Diurnal Regulated Biological Processes and Key Pathways in Rice Flag Leaves and Seedling Leaves
Source: PLoS One. 2011 Mar 2;6(3):e17613. doi: 10.1371/journal.pone.0017613 (PMC3047585; doi:10.1371/journal.pone.0017613)

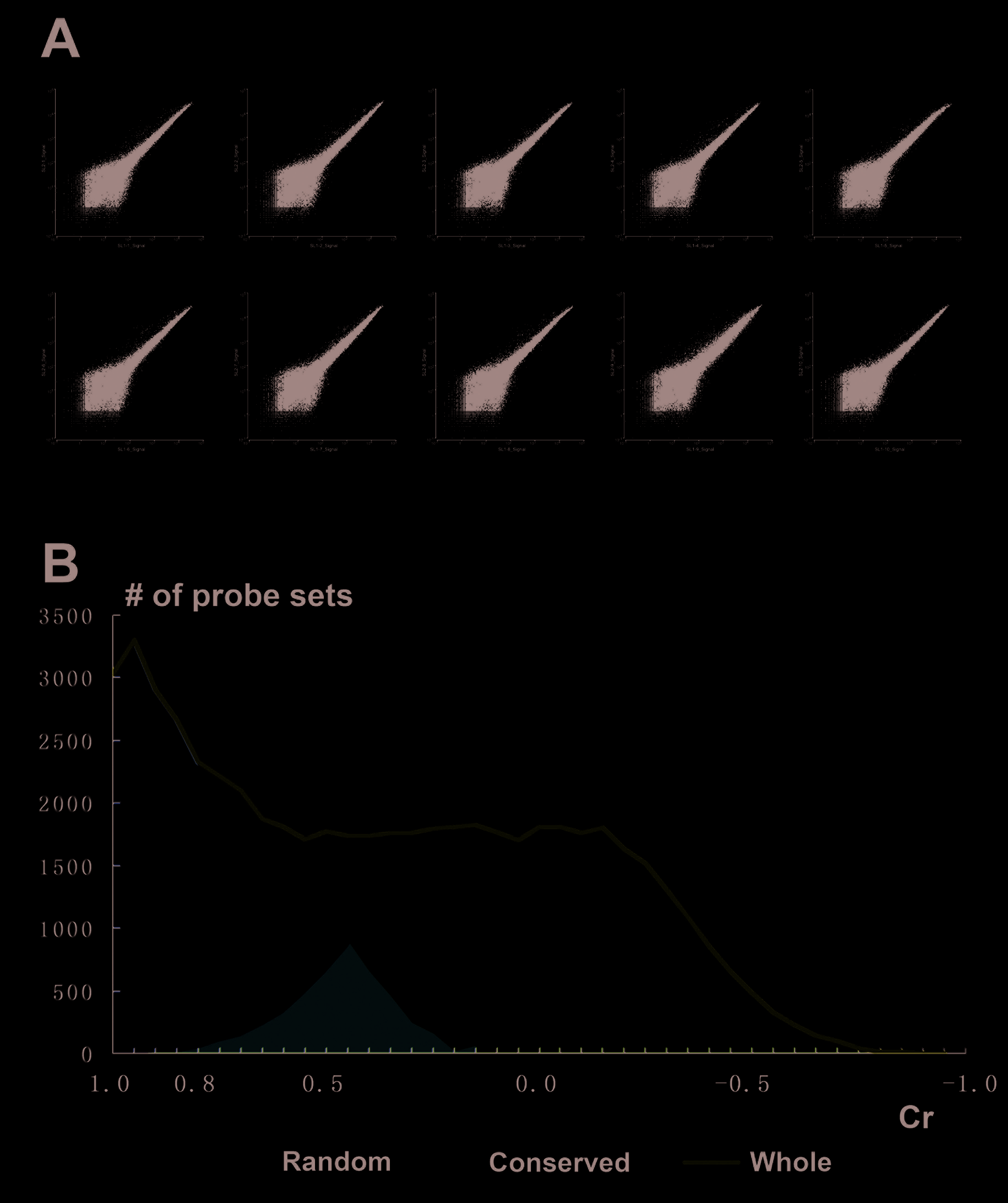

Supplement: Figure S1 — Expression profiles between the biological replicate samples of seedling leaves. A: Pair-wise scatter plots for the raw probe-set intensity data across all time points. B: The number distribution of all probe sets based on the correlation coefficient (Cr) across the time series between the biological replicate samples (including file name, file format, name and URL link of appropriate viewer if format is unusual). (TIF) [file pone.0017613.s001.tif]
